# Impact of anti-tuberculosis treatment on hematological parameters in newly diagnosed tuberculosis patients at Jimma town: a longitudinal prospective study

**DOI:** 10.1097/MS9.0000000000001084

**Published:** 2023-07-17

**Authors:** Birhane Reta, Abdurehman E. Mohammed, Girum Tesfaye Kiya, Wondimagegn Adissu, Tilahun Y. Shenkute

**Affiliations:** a Department of Medical Laboratory Sciences, Jimma University, Jimma, Ethiopia; b Department of Medical Laboratory Sciences, Wollo University, Dessie, Ethiopia

**Keywords:** anemia, anti-tuberculosis drugs, hematological profile, leucopenia, tuberculosis

## Abstract

Tuberculosis (TB) is a significant public health problem affecting one-third of the world’s population. In 2021, 1.6 million people died from TB. TB is the 13th leading cause of death and the second leading cause of infectious death after coronavirus disease 2019. Most anti-TB drugs affect hematological parameters in patients.

**Methods:**

This longitudinal prospective study was conducted from 03 January to 30 December 2019. Patients who completed a course of TB treatment were candidates for analysis. Sputum and blood samples were collected from each study participant and analyzed by the Gene X-pert machine and a HumaCount 30 hematology analyzer (Human GmbH). SPSS version 20 and R programming software version 4.2.3 were used for data analysis. Friedman’s test was used to assess statistical significance. *P*-values ​​less than 0.05 were considered statistically significant.

**Results:**

A total of 148 patients who completed the course of TB treatment correctly were a candidate for final analysis. Ninety-one (61.5%) study participants were male; the median age was 27.6±9.8 years. Moreover, most of the study participants (84.4%) had pulmonary TB. Most of the hematological parameters had changed in the phases of TB treatment. After anti-TB treatment, there is a significant difference in hematological parameters in red blood cell count, hemoglobin concentration, hematocrit percentage, platelet count, and white blood cell count.

**Conclusion:**

Anemia and leucopenia are the most significant problems after TB treatment. Regular checking of these parameters is essential for the patient.

## Introduction

Tuberculosis (TB) is an infectious disease caused by *Mycobacterium tuberculosis* and kills millions of people each year around the globe^1^. TB is mainly a disease of the lungs, but it can affect any body organ^2^. *Mycobacterium tuberculosis* is a rod-shaped nonspore-forming, aerobic bacterium spread by tiny airborne droplets, called droplet nuclei, generated by the coughing, sneezing, talking, or singing of a person with pulmonary TB^3^.

TB is a significant public health problem affecting one-third of the world’s population^4^. In 2021, 1.6 million people died because of TB. TB is the 13th most common cause of mortality and the 2nd most common infection killer after coronavirus disease 2019^1^. Ethiopia ranked seventh in the world for TB burden and third in Africa, with an estimated incidence of 378 new cases per 100 000 persons^5,6^.

The standard treatment regimen for drug-susceptible TB includes an initial intensive phase (2 months) of treatment consisting of rifampicin, isoniazid, pyrazinamide, and ethambutol in combination. The continuation phase consists of rifampicin and isoniazid for an additional 4 months of treatment^7,8^. Most anti-TB drugs affect the hematological parameters of the patient^9–14^. The common adverse effects of anti-TB drugs on patients are anemia and thrombocytopenia^15,16^. Minimizing the impact of anti-TB treatments has a significant role in preventing a hematological change in the patient. In Ethiopia, there is limited information on the impact of anti-TB treatment on hematological parameter changes. This study aims to evaluate the effects of anti-TB therapy on some hematologic parameters in newly diagnosed TB patients.

## Methods

### Study design

A longitudinal prospective study was conducted from 03 January to 30 December 2019. The study population was bacteriologically or clinically confirmed new TB-positive patients attending governmental health institutions during the study period. Patients with known chronic disease, HIV-positive patients, pregnant women, patients who had observed signs and symptoms of hemiparasite, intestinal parasite-positive patients, retreatment cases, and patients with any other treatment exposure were excluded from the study.

The sample size was calculated by using the single population proportion formula. The prevalence of anemia for the population, which was 0.72^9^, 95% CI, and a 5% of margin of error, was used to calculate the sample size. The calculated sample size was 310. A convenient sampling technique was used to collect the needed samples. We conducted this study following the strengthening the reporting of cohort, cross-sectional and case–control studies in surgery (STROCSS) 2021 recommendations^17^.

### Data collection procedure

The sociodemographic and clinical data were collected with a pretested structured questionnaire. All TB-suspected patients with signs and symptoms of TB were the source populations. All the clinical sample collection and laboratory analyses were done following SOP strictly.

### Blood specimen collection and laboratory analysis

Four milliliters of blood samples were collected from each study participant in each treatment phase. The three blood samples were collected in weeks 9 and 25 before treatment initiation. The collected blood sample was analyzed within 8 h of collection with a HumaCount 30^TS^ Hematology analyzer (Human GmbH).

### Sputum sample collection and diagnosis of tuberculosis

Sputum samples were collected from TB-suspected patients and analyzed with a Gene X-pert machine (Cepheid, GLI, USAID). In some health institutions, TB was diagnosed using smear-microscopy stained with Ziehl–Neelsen (ZN) stain, and results were reported by following the WHO TB reporting methods^18^.

### Sample collection and diagnosis of extrapulmonary tuberculosis

The extrapulmonary TB was diagnosed by collecting fluid from the affected area by physicians, stained with ZN stain, and examined under the 100× oil immersion objective with bright field microscopy. Moreover, the clinical diagnosis of TB was made by physicians.

### Follow-up data collection

Follow-up data were collected from each participant using a questionnaire. Weekly follow-up information was collected, including the symptoms of any illness and other treatment exposure in the previous week. For the continuous phase, the correct treatment uptake was checked through telephone and weekly contact with the patients. Sputum and blood specimens were collected from each study participant.

### Data analysis

All the data from the questionnaires and laboratory results were checked for completeness, cleaned, entered into Epi-data version 4.4.1, and exported to SPSS software version 20. Checking for normality was done using the Kolmogorov–Smirnov (K-S) test, and a *P*-value>0.05 was considered normally distributed. The statistical significance of the association and the graphs were done by R programming software version 4.2.3^19^. To assess the statistical significance associations, the Friedman test was used. *P*-values less than 0.05 were considered statistically significant.

## Results

### Sociodemographic characteristics of the study participants

Three hundred and ten newly diagnosed TB patients were involved in this study. One hundred sixty-two patients were excluded from the analysis because of different reasons such as 98 patients had other treatment exposure during treatment, 7 TB patients did not utilize anti-TB treatment correctly, 12 TB patients had smear-positive after the intensive phase of treatment, 44 TB patients were unwilling to give follow-up information and 1 patient died during treatment.

Only 148 newly diagnosed TB patients who completed the course of TB treatment correctly were a candidate for analysis. Of them, 91 (61.5%) were male, and the mean age of study participants was 27.6±9.8 years ranging from 7 to 70 years. Fifteen (10.1%) of the study participants were under 18. Most 134(90.5%) of the study participants were urban dwellers and attended primary school 61 (41.2%) (Table 1).

**Table 1:** Sociodemographic characteristics of newly diagnosed tuberculosis patients (*n*=148) public health facilities 2019.

	Sex		
Variables	Male (%)	Female (%)	Total (%)
Age (years)			
5–17	12 (8.1)	3 (2.03)	15 (10.1)
18–59	75 (50.67)	54 (36.5)	129 (87.2)
>60	4 (2.7)	0 (0)	4 (2.7)
Residence			
Urban	82 (55.4)	52 (35.1)	134 (90.5)
Rural	9 (6.1)	5 (3.37)	14 (9.5)
Marital status			
Married	45 (30.4)	33 (22.3)	78 (52.7)
Unmarried	46 (31.1)	24 (16.2)	70 (47.3)
Educational status			
Illiterate	16 (10.81)	19 (12.83)	35 (23.64)
Primary school	38 (25.67)	23 (15.54)	61 (41.2)
Secondary school	19 (12.83)	15 (10.13)	34 (22.96)
University	18 (12.16)	0 (0)	18 (12.16)

### Clinical characteristics of newly diagnosed tuberculosis patients

Regarding the clinical status, most of the study participants had night sweat (98.6%), cough (92.5%), fever (72.9%), weight loss (93.3%), chest pain (82.4%), and shortness of breath (84.4%). TB patients’ clinical and laboratory data showed that 125 (84.4%) and 23 (15.6%) cases were pulmonary and extrapulmonary TB cases, respectively (Table 2).

**Table 2:** Clinical characteristics of newly diagnosed tuberculosis patients (*n*=148), 2019.

	Sex		
Variables	Male (%)	Female (%)	Total (%)
Cough			
Yes	83 (56.1)	54 (36.5)	137 (92.6)
No	8 (5.4)	3 (2.0)	11 (7.4)
Fever			
Yes	61 (41.2)	47 (31.8)	108 (73)
No	30 (20.3	10 (6.7)	40 (27)
Weight loss			
Yes	84 (56.8)	54 (36.5)	138 (93.3)
No	7 (4.7)	3 (2)	10 (6.7)
Night sweat			
Yes	90 (60.83)	56 (37.83)	146 (98.63)
No	1 (0.67)	1 (0.67)	2 (1.34)
Hemoptysis			
Yes	1 (0.67)	1 (0.67)	2 (1.34)
No	90 (60.83)	56 (37.83)	146 (98.66)
Chest pain			
Yes	71 (47.97)	51 (34.5)	122 (82.47)
No	20 (13.5)	6 (4)	26 (17.5)
Shortness of breath			
Yes	75 (50.7)	50 (33.8)	125 (84.5)
No	16 (10.8)	7 (4.7)	23 (15.5)
Types of TB			
Pulmonary	77 (52)	48 (32.4)	125 (84.4)
Extrapulmonary	14 (9.5)	9 (6.1)	23 (15.6)

### Hematological parameters among TB patients in the different phases of tuberculosis treatment

#### Red blood cells

Most of the study participants had a normal red blood cells (RBC) counts in all the phases of TB treatment. However, some study participants had low RBC counts: before initiation of treatment, 29 (21.6%); after intensive phases of treatment, 16 (10.8%); and after completion of treatment, 32 (21.6%), respectively. On the other hand, before the initiation of treatment, 4 (2.7%) of TB patients had a high RBC count (Table 3).

**Table 3:** The proportion of hematological parameters with low, normal, and high values of the TB patients (*n*=148) in the different phases of tuberculosis treatment, 2019.

Parameters	Before initiation of treatment (%)	After an intensive phase of treatment (%)	After completion of treatment (%)	Reference interval (20)
RBC×10^12^/l				
Low	29 (19.6)	16 (10.8)	32 (21.6)	Male=4.26–6.68×10^12^/l (A) Female=4.02–6.15×10^12^/l (A) Male=4.06–6.57×10^12^/l (C) Female=4.32–5.63×10^12^/l (C)
Normal	115 (77.7)	132 (89.2)	116 (78.4)	
High	4 (2.7)	0 (0)	0 (0)	
WBC×10^9/^/l				
Low	0 (0)	1 (0.7)	12 (8.1)	Male=3.31–11.62×10^9^/l (A) Female=3.24–10.05×10^9^/l (A) Male=4.04–11.72×10^9^/l (C) Female=3.74–11.42×10^9^/l (C)
Normal	122 (82.4)	147 (99.3)	136 (91.9)	
High	26 (17.6)	0 (0)	0 (0)	
Lymphocyte×10^9^/l				
Low	4 (2.7)	25 (16.9)	1 (0.7)	Male=1.1–3.84×10^9^/l (A) Female=1.2–3.98×10^9^/l (A) Male=1.5–4.25×10^9^/l (C) Female=1.41–4.47×10^9^/l (C)
Normal	139 (93.9)	117 (79.1)	146 (98.6)	
High	5 (3.4)	6 (4.1)	1 (0.7)	
Neutrophil×10^9^/l				
Low	0 (0)	0 (0)	4 (2.7)	Male=1.01–7.22×10^9^/l (A) Female=1.08–6.69×10^9^/l (A) Male=1.26–7.39×10^9^/l (C) Female=1–6.99×10^9^/l (C)
Normal	98 (66.2)	143 (96.6)	144 (97.3)	
High	50 (33.8)	5 (3.4)	0 (0)	
Hemoglobin (g/dl)				
Low	67 (45.3)	21 (14.2)	35 (23.6)	Male=12.6–18.7 g/dl (A) Female=12.3–17.8 g/dl (A) Male=12.0–19.6 g/dl (C)
Normal	77 (52)	127 (85.8)	113 (76.4)	
High	4 (2.7)	0 (0)	0 (0)	
Hematocrit (%)				Female=11.6–15.9 g/dl (C)
Low	49 (33.1)	32 (21.6)	33 (22.3)	Male=36.72–54.48% (A) Female=36.86–51.59% (A) Male=35.6–55.2% (C) Female=35.97–46.92% (C)
Normal	95 (64.2)	113 (76.4)	110 (74.3)	
High	4 (2.7)	3 (2)	5 (3.4)	
MCH (pg)				
Low	18 (12.2)	5 (3.4)	1 (0.7)	Male=24.86–32.84 pg (A) Female=26.3–33.58 pg (A) Male=25.18–31.05 pg (C) Female =25.08–30.8 pg (C)
Normal	130 (87.8)	143 (96.6)	129 (87.2)	
High	0 (0)	0 (0)	18 (12.2)	
MCHC (g/l)				
Low	56 (37.8)	37 (25)	44 (29.7)	Male=32.6–36.5 g/l (A) Female=32–36 g/l (A) Male=32.1–36.2 g/l (C) Female=32.07–35.44 g/l (C)
Normal	81 (54.7)	74 (50)	100 (67.6)	
High	11 (7.4)	37 (25)	4 (2.7)	
MCV (FL)				
Low	19 (12.8)	14 (9.5)	4 (2.7)	Male=74.8–93.9 FL (A) Female=77.3–98.8 FL (A) Male=75.03–93.01 FL (C) Female=74.51–91.08 FL (C)
Normal	124 (83.8)	122 (82.4)	113 (76.4)	
High	5 (3.4)	12 (8.1)	31 (20.9)	
RDW-CV (%)				
Normal	98 (66.2)	98 (66.2)	95 (64.2)	Male=12.4–17.5% (A) Female=12.4–15.59% (A) Male=12.70–16.07% (C) Female=12.30–15.97% (C)
High	20 (13.5)	46 (31.1)	2 (1.4)	
Low	4 (2.7)	1 (0.7)	0 (0)	
Platelet×10^9^/l				
Low	4 (2.7)	1 (0.7)	0 (0)	Male=164–403×10^9^/l (A) Female=202.2–444.5×10^9^/l (A) Male=158.5–469.9×10^9^/l (C) Female=197.7–460.4×10^9^/l (C)
Normal	68 (45.9)	84 (56.8)	125 (84.5)	
High	76 (51.4)	63 (42.6)	23 (15.5)	

A, adult; C, children.

#### White blood cells

Most TB patients had normal white blood cells (WBC) count in all phases of TB treatment. Before initiation of treatment, 26 (17.6%) of the TB patients had leukocytosis, but after the intensive phase of treatment and after completion of treatment, the WBC count reduced to normal. After completion of treatment, 12 (8.1%) of the TB patients showed low WBC counts. Twenty-six patients had leukocytosis before TB treatment initiation. They all had improved to an average white blood cell count after TB initiation. There were no neutropenic patients before and after the intensive phase of treatment, but 4 (2.7%) TB patients had neutropenia after the completion of treatment (Table 3).

#### Hemoglobin

In this study, 67(45.3%), 21(14.2%), and 35(23.6%) of the TB patients showed low hemoglobin (Hgb) concentrations before initiation of treatment, after intensive phases of treatment, and after completion of treatment, respectively (Table 3).

#### Red cell indices

The proportion of TB patients with a high mean corpuscular volume (MCV) value was slightly raised after the completion of treatment, 31 (20.9%), compared with before treatment 5 (3.4%) and after the intensive phase of treatment 12 (8.1%). The proportion of TB patients with low MCV was slightly reduced after the second month of treatment, 14 (9.5%), and after completion of treatment, 4 (2.7%) compared with before the initiation of treatment, 19 (12.8%) (Table 3).

#### Platelet

The proportion of TB patients with low platelet (PLT) count was slightly reduced after 2 months, 1 (0.7%), and after 6 months of treatment, compared with before treatment 4 (2.7%). TB patients had a high PLT count of 76 (51.4%) before treatment, which was slightly reduced after 2 months of treatment 63 (42.6%) and after 6 months of treatment 23 (15.5%), respectively (Table 3).

### Hematological parameters change in tuberculosis patients before initiation of treatment, after the intensive phase of treatment, and after completion of treatment (Tx)

**Figure 1:**
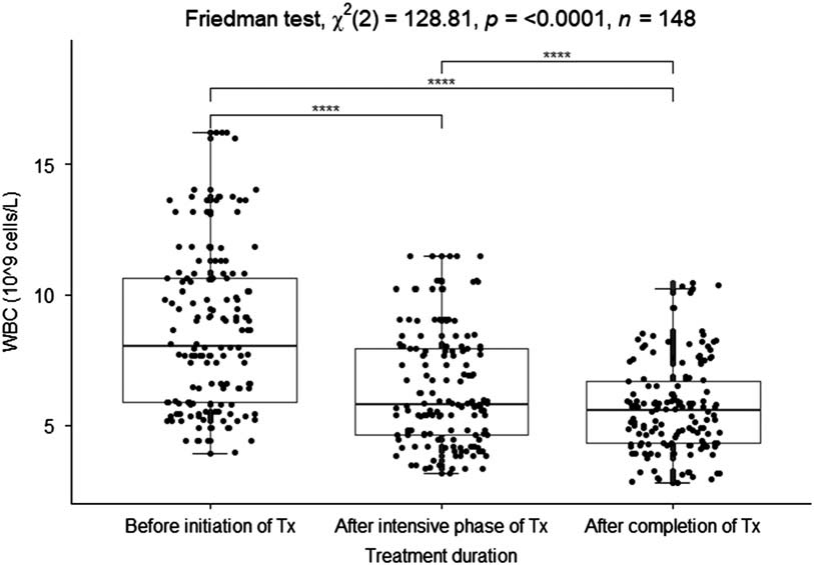
Boxplot of the median white blood cells count before initiation, after the intensive phase of treatment, and after completion of treatment. *P<0.05, **P<0.01, ***P<0.005, ****P<0.0001 were considered significant.

Most of the hematological parameters had changed during the phases of TB treatment. There was a high WBC (8.04×10^9^ cells/l) before the initiation of treatment, and the number gradually reduced in the intensive phase of treatment (5.81×10^9^ cells/l) and after completion of treatment (5.55×10^9^ cells/l) (reference interval, male=3.31–11.62×10^9^/l adult (A), female=3.24–10.05×10^9^/l (A), male=4.04–11.72×10^9^/l children (C), female=3.74–11.42×10^9^/l (C)^20^). There was a strong significant association among the median WBC count before initiation of treatment, after the intensive phase of treatment, and after completion of treatment (Friedman test, *X* ^2^(2)=128.81, *P*<0.0001). There was a strong and significant association between the median WBC count before initiation of treatment and after completion of Treatment (*P*<0.0001). Moreover, there was a strong significant association between the median WBC count before the initiation of treatment and after the intensive phase of treatment (*P*<0.000) (Fig. 1).

#### Hemoglobin

**Figure 2:**
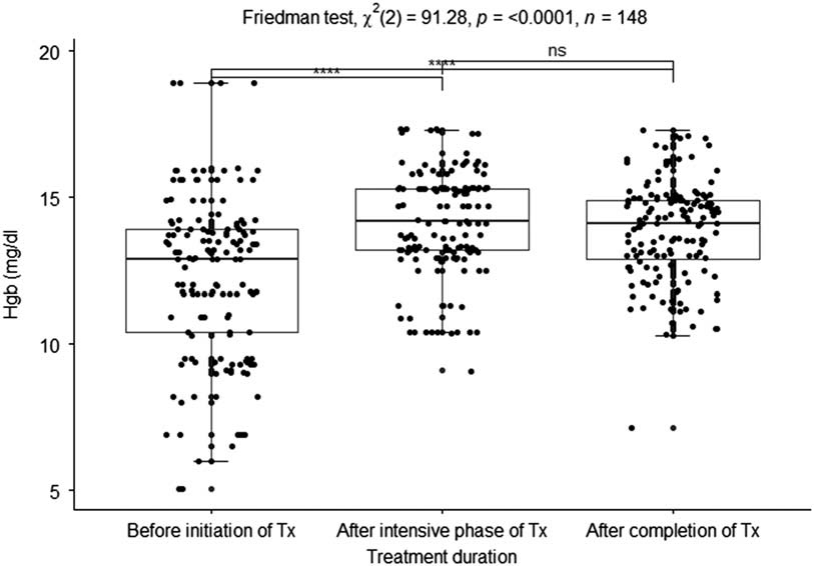
Boxplot of the median hemoglobin concentration before initiation of treatment, after the intensive phase, and after treatment completion. *P<0.05, **P<0.01, ***P<0.005, ****P<0.0001 were considered significant.

During the initial phase of treatment, the median Hgb was low (12.9 mg/dl). However, after the intensive phase and completion of TB treatment, the median Hgb was increased to 14.2 mg/dl and 14.1 mg/dl, respectively (reference interval, male=12.6–18.7 g/dl (A), female=12.3–17.8 g/dl (A), male=12.0–19.6 g/dl (C), female=11.6–15.9 g/dl (C)^20^). Hgb was increased in both the intensive phase of treatment and after the completion of treatment. There were significant solid associations among the median Hgb concentrations in the three phases of TB treatment (Friedman test, *X* ^2^ (2)=91.28, *P*<0.0001). Significant solid associations existed between the median Hgb concentration of TB treatment’s initial and intensive phase (*P*<0.0001). Moreover, the median of Hgb in the initial phase of treatment and after completion of treatment had significant solid associations (*P*<0.0001). Unfortunately, the median Hgb concentrations of the intensive phase of treatment and after completion of TB treatment had no significant associations (*P*=0.1546) (Fig. 2).

#### Platelet

**Figure 3:**
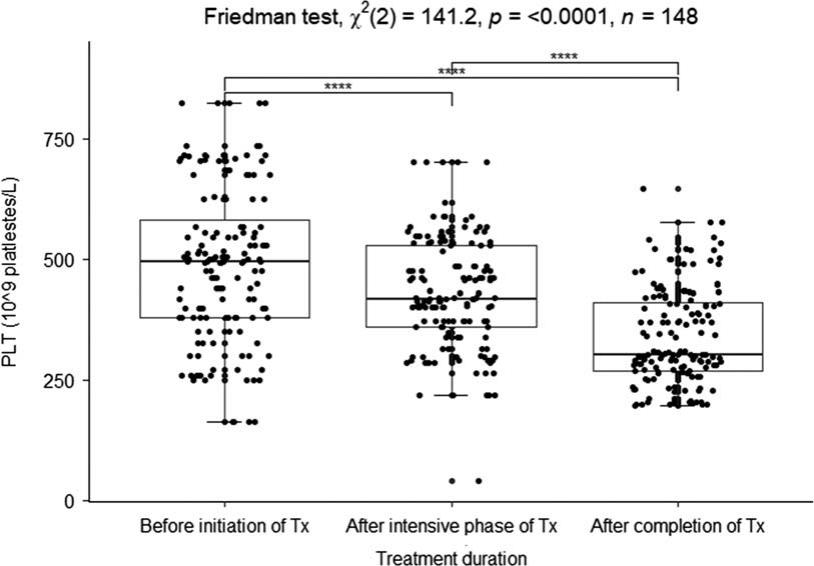
Boxplot of the median platelet before initiation of treatment, after the intensive phase, and after treatment completion. *P<0.05, **P<0.01, ***P<0.005, ****P<0.0001 were considered significant.

There was a high PLT count before the initiation of treatment, which counts about 494×10^9^ platlestes/l, then the PLT count decreased after the intensive phase of treatment (417×10^9^ platlestes/l and completion of treatment (303×10^9^ platlestes/l) (reference interval, male=164–403×10^9^/l (A), female=202.2–444.5×10^9^/l (A), male=158.5–469.9×10^9^/l (C), female=197.7–460.4×10^9^/l (C)^20^). There was a strong and significant association among the median values of PLTs in the phases of TB treatment (Friedman test, *X* ^2^=141.2, *P*<0.0001) (Fig. 3).

#### Hematocrit

**Figure 4:**
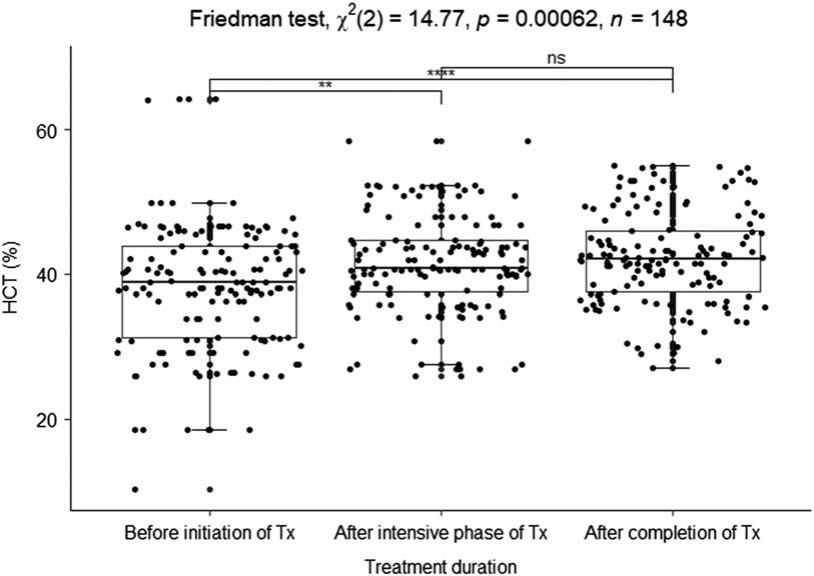
Boxplot of the median hematocrit before initiation of treatment, after the intensive phase, and after treatment completion. *P<0.05, **P<0.01, ***P<0.005, ****P<0.0001 were considered significant. ns, no significant association.

The median hematocrit (HCT) was increased in the intensive phase (40.8%) and after completion of TB treatment (42.05%) than in the initial phase of TB treatment (38.89%) (reference interval, male=36.72–54.48% (A), female=36.86–51.59% (A), male=35.6–55.2% (C), female=35.97–46.92% (C)^20^). The TB treatment improves the HCT percentage. Significant associations were among the median HCT percentage in the three phases of TB treatment (Friedman test, *X* ^2^(2)=14.77, *P*=0.0006). There was a strong and significant association between the median HCT before initiation of treatment and after completion of Treatment (*P*=0.0005). There were slightly significant associations between the median HCT before the initiation of treatment and after the intensive phase of treatment (*P*=0.0373). The median HCT after the intensive phase of treatment and after completion of treatment had no significant associations (*P*=0.6030) (Fig. 4).

#### Mean corpuscular volume

**Figure 5:**
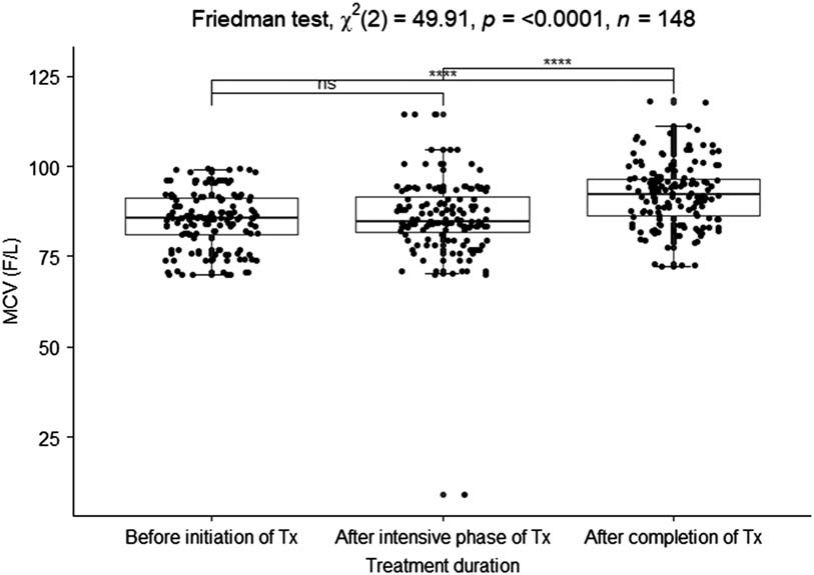
Boxplot of the median mean corpuscular volume before initiation of treatment, after the intensive phase, and after treatment completion. *P<0.05, **P<0.01, ***P<0.005, ****P<0.0001 were considered significant. Tx, treatment.

The median MCV before initiation, after the intensive phase, and after treatment completion were 85.6, 84.6, and 92.2 FL, respectively (reference interval, male=74.8–93.9 FL (A), female=77.3–98.8 FL (A), male=75.03–93.01 FL (C), female=74.51–91.08 FL (C)^20^). There was an enormously significant association among the median of the MCV in the phases of TB treatment (Friedman test, *X* ^2^=49.91, *P*<0.0001). There was no significant association between the median before the initiation of treatment and after the intensive phase of treatment (*P*=0.6667). There was a strong significant association between the median of the MCV after the intensive treatment phase and after treatment completion (*P*<0.0001) (Fig. 5).

## Discussion

One of the adverse effects of anti-TB drugs is affecting some of the hematological parameters^21^. In this study, the TB patients’ median WBC before initiation of anti-TB treatment, after the intensive treatment phase, and after treatment completion were 8.04×10^9^ cells/l, 5.81×10^9^ cells/l, and 5.5×10^9^ cells/l, respectively. The increased median value of leukocyte count before anti-TB treatment might be due to the increased neutrophil count in TB patients before the initiation of treatment. This result agrees with the study done in Kerala, India^22^, and Maharashtra, India^23^, and is discordant with the study conducted in Karnataka, India^24^. The variation in significance level between the results might be due to incomplete data of TB patients until the last course of treatment and a sample size difference.

Neutropenia was observed in 4 (2.7%) TB patients after the completion of treatment in the current study. Our result agrees with the study done in the USA^25^. The difference might be because of anti-TB treatment. The current study found that TB patients’ median RBC, HGB, and HCT values showed a significant difference after treatment administration compared to before initiating anti-TB treatment. Our result agrees with the study done in Karnataka, India.^24^, and concordant with the study done in Gondar, Ethiopia^9^. This might be due to differences in the duration of treatment and sample size differences.

In the present study, 67 (45.3%) TB patients had anemia before initiating anti-TB treatment. TB patients with anemia after the intensive phase of treatment were reduced to 21 (14.2%). Our result agrees with the study done in Karnataka, India^12^, and Korea^26^. In our study, there were 4 (2.7%) thrombocytopenic patients before initiating anti-TB treatment. This result agrees with a study in Karnataka, India^22^.

## Conclusion

The proportion of TB patients with leukocytosis, agranulocytosis, thrombocytopenia, and thrombocytosis after completion of treatment decreased compared to the proportion of TB patients before anti-TB treatment. Anemia and leucopenia are the main findings after the completion of anti-TB treatment. After treatment, the decreased proportion of hematological abnormalities such as leukocytosis, granulocytosis, thrombocytopenia, and thrombocytosis reflects that these parameters can indicate a good treatment response. However, anemia and leucopenia do not improve throughout treatment, highlighting the necessity of continuously checking these parameters. There are very few articles published in this area, and this study will give information on the adverse effect of anti-TB drugs in hematological profiles.

### Limitations of the study

Because of the study’s nature and cost, a small sample size was included in the final analysis.

We followed each patient for only 6 months and assessed the hematological parameters; the hematological parameters status in post-anti-TB treatment is not known. Finally, because of the complexity of the bone marrow procedure, a bone marrow biopsy is not taken from patients to compare the result with the hematological profiles.

## Ethical approval

Ethical approval for this study (Institution Review Board Number HRPr00743/19) was provided by our university’s Institution Review Board (IRB) on 21 February 2019.

## Consent

Written informed consent was taken from each study participant.

## Sources of funding

Jimma University funded the research.

## Author contribution

A.E.M.: formal analysis, writing original draft, and reviewing and editing; B.R.: conceptualization, methodology, and reviewing and editing the manuscript; G.T.: investigation, project administration and reviewing and editing the manuscript; W.A.: data curation, investigation and reviewing and editing the manuscript; T.Y.S.: supervision, validation and reviewing and editing the manuscript.

## Conflicts of interest disclosure

There is no conflict of interest.

## Research registration unique identifying number (UIN)

Name of the registry: not applicable.
Unique Identifying number or registration ID: not applicable.
Hyperlink to your specific registration (must be publicly accessible and will be checked): not applicable.

## Guarantor

Abdurehman Eshete Mohammed.

## Data availability statement

Data is available upon reasonable request.

## Provenance and peer review

Not commissioned, externally peer-reviewed.

## Acknowledgements

The authors thank Jimma University for the research grant and are grateful to all the study participants.
